# Identification of highly related references about gene-disease association

**DOI:** 10.1186/1471-2105-15-286

**Published:** 2014-08-25

**Authors:** Rey-Long Liu, Chia-Chun Shih

**Affiliations:** Department of Medical Informatics, Tzu Chi University, Hualien, Taiwan

**Keywords:** Gene-disease association, Reference ranking, Conclusiveness of information, Richness of information, Focus of information

## Abstract

**Background:**

Curation of gene-disease associations published in literature should be based on careful and frequent survey of the references that are highly related to specific gene-disease associations. Retrieval of the references is thus essential for timely and complete curation.

**Results:**

We present a technique CRFref (Conclusive, Rich, and Focused References) that, given a gene-disease pair < *g*, *d*>, ranks high those biomedical references that are likely to provide *conclusive*, *rich*, and *focused* results about *g* and *d*. Such references are expected to be highly related to the association between *g* and *d*. CRFref ranks candidate references based on their scores. To estimate the score of a reference *r*, CRFref estimates and integrates three measures: *degree of conclusiveness*, *degree of richness*, and *degree of focus* of *r* with respect to < *g*, *d*>. To evaluate CRFref, experiments are conducted on over one hundred thousand references for over one thousand gene-disease pairs. Experimental results show that CRFref performs significantly better than several typical types of baselines in ranking high those references that expert curators select to develop the summaries for specific gene-disease associations.

**Conclusion:**

CRFref is a good technique to rank high those references that are highly related to specific gene-disease associations. It can be incorporated into existing search engines to prioritize biomedical references for curators and researchers, as well as those text mining systems that aim at the study of gene-disease associations.

## Background

A gene is said to be associated with a disease if its certain type of alteration is more frequent in people with the disease. For the purposes of disease diagnosis, prognosis, and therapy, much research effort was devoted to identifying gene-disease associations. A large amount of gene-disease associations have been reported in biomedical literature and search engines have been developed to retrieve the literature (e.g., medical genetics search services provided by PubMed^*a*^). To facilitate knowledge sharing and further research, the gene-disease associations reported in the literature need to be curated, and hence several online databases of the gene-disease associations have been built and maintained. Typical examples of such databases are Genetic Home Reference (GHR)^*b*^ and Online Mendelian Inheritance in Human (OMIM)^*c*^.

However, it is quite difficult to curate gene-disease associations in a complete and timely manner, mainly due to two reasons: (1) a huge and ever-growing amount of biomedical references need to be searched for the large number of possible gene-disease pairs, and (2) curation of even a single gene-disease association needs to be based on a careful survey of biomedical literature (e.g., for each association the curators of GHR need to carefully find and check multiple articles to exclude unproven or controversial information^*d*^). Therefore, a large number of biomedical experts are involved in the curation task (e.g., hundreds of experts in GHR review information for the curation^*e*^), and the curators need to routinely spend much effort maintaining the association database (e.g., OMIM updates its database on a daily basis^*f*^, and GHR conducts several updates in each week^*g*^). New research findings thus often take time to be curated from biomedical literature [1–4].

### Problem definition and main contribution

In this paper, we present a technique CRFref (Conclusive, Rich, and Focused References) that, given a gene *g* and a disease *d*, ranks high those biomedical references that are likely to provide *conclusive*, *rich*, and *focused* results about *g* and *d*. We believe that such references are highly related to the association between *g* and *d*, and hence ranking them high can help expert curators to curate gene-disease associations in a more complete and timely manner.

Technically, candidate references are ranked based on their scores. To estimate the score of a reference *r*, CRFref estimates and integrates three measures: *degree of focus*, *degree of richness*, and *degree of conclusiveness* of *r* with respect to the given gene-disease pair < *g*, *d*>. Automatic estimation and integration of the three measures are challenging. The degree of conclusiveness is measured by considering how *g* and *d* appear in the “concluding” parts of *r* (i.e., title and ending part of *r*), which are those parts that are more likely to convey the conclusion of *r*. If both *g* and *d* tend to appear in the concluding parts, the conclusion of *r* may be related to the association between *g* and *d*. For the degree of richness, CRFref considers how *r* mentions different kinds of genes and diseases that are neither *g* nor *d*. We expect that *r* might provide rich information about < *g*, *d* > if there are many such genes and diseases in *r*. Note that the expectation is reasonable only when *r* has provided *conclusive* information about < *g*, *d*>, since in that case the existence of the information about other genes and diseases can indicate that the author of *r* has tried to provide more information about < *g*, *d* > in *r*. Therefore, it may be helpful to integrate the degree of conclusiveness and the degree of richness. Finally, for the degree of focus, CRFref considers how *r* provides *conclusive* information about many genes and diseases that are *neither g nor d*. If *r* does not provide such information, it should be focused on < *g*, *d* > (i.e., the conclusive information of *r* is not diluted), and hence should have a larger score.

### Related work

#### Prediction and extraction of gene-disease associations

Previous techniques that supported the analysis of gene-disease associations often fell into two types: (1) those that *predict* possible gene-disease associations, and (2) those that *extract* the evidence about specific gene-disease associations. The former aimed at suggesting possible gene-disease associations for biomedical researchers to explore and verify; while the latter aimed at retrieving from biomedical references the evidence about specific genes and diseases so that researchers can base their new research on the current evidence that has been found and published.

A survey of the prediction techniques can be found in [5]. Typical resources employed by the prediction techniques included chromosomal locations of the candidate genes [6], anatomical ontology [7], gene ontology [8], protein-protein interaction networks (e.g., employed by the GeneWanderer system^*h*^), indirect but related evidence about a gene *g* and a disease *d* reported in the literature [8], overlap of the index terms of references related to *g* and *d* [9], co-occurrence of the concepts related to *g* and *d* in literature [4, 6, 10], published interactions between *g* and those genes that are related to *d* [1], and occurrence of *g* and *d* in overlapping clusters of literature [11]. Instead of predicting possible gene-disease associations, CRFref aims at ranking high those biomedical references that are highly related to specific gene-disease pairs. The reference ranking task is to support the analysis and curation of the associations that have been reported in literature. From this point of view, CRFref is related to those extraction techniques that extract information about genes and diseases in literature.

However, performance of the existing association extraction techniques was often limited, even though various language processing and learning techniques have been developed. Some studies reported that precision of the extraction was about 0.65 [2, 12], and it was even lower in some cases [1, 3, 13]. The improvement of the precision rate was often at the cost of deteriorating the recall rate [14]. The performance of the extraction systems may be even worse when the syntactic, semantic, anaphoric, and discourse structures of the references are beyond the scope of technical considerations of the systems. Therefore, although the extraction techniques are still being refined (e.g., employing more association-indicative terms in the extraction [15]), previous studies have turned to focus on computer-aided curation so that the quality of the curation can be promoted (e.g., manually confirming and enriching the associations between biomedical entities [3]). CRFref is a technique to support the computer-aided curation. Given highly related references recommended by CRFref, curators can check and extract gene-disease associations in the references, and hence guarantee the quality of the curated gene-disease associations. Reference recommendation is essential for the curation of biomedical information [16], and it has been a fundamental component in the systems that supports the curation (e.g., [17, 18]). CRFref is the first system that recommends those references that provide conclusive, rich, and focused results about specific gene-disease associations. It can contribute to the existing extraction techniques as well. The extraction techniques can extract gene-disease associations from the highly related references recommended by CRFref.

#### Text ranking technology

CRFref ranks references based on how they provide conclusive, rich, and focused results about specific gene-disease pairs. Therefore, it is technically related to those previous studies that aimed at text ranking technology and its application to biomedicine.

Since text ranking is an essential function of search engines, previous information retrieval studies have developed many ranking techniques. Given a query, the rankers assigned a score to each text, and the texts were ranked based on the scores. Typical factors employed to estimate the score of a text *r* included the frequency of each query term appearing in *r*, the weight of the query term, and the length of *r* (for a list of the ranking techniques, the reader is referred to [19]). Therefore, *r* tends to have a larger score if those query terms with higher weights appear frequently in *r*. Machine learning approaches were also developed to integrate multiple rankers [20–24]. However, the rankers did not consider the degrees of conclusiveness, richness, and focus of *r* with respect to the query, which consists of a gene and a disease. We will show that the degrees of conclusiveness, richness, and focus are particularly useful in supporting the manual identification of those references that are highly related to gene-disease associations.

*Proximity* of query terms in the text was also employed to improve the previous rankers, based on the expectation that if query terms appear in a nearby area in a text *r*, the score of *r* should be amplified as *r* is likely to be relevant to the query [25–28]. Proximity of a gene and a disease in a reference was also employed to extract the sentences that provided the evidence about the association between the gene and the disease [13]. We will show that CRFref outperforms two state-of-the-art proximity-based techniques in ranking high those references that biomedical experts believe to be highly related to the associations between the genes and the diseases.

*Positions* and *frequencies* of terms in the references were employed to identify relevant biomedical information as well. To extract the information about the functions of genes, information systems were developed to extract text passages (e.g., sentences) from a given reference by selecting those passages that appear at certain positions in the reference [29, 30]. To automatically classify sentences in a reference into the categories about evidence-based medicine (EBM), positions of the terms in the reference were also shown to be good features for the classifiers [31, 32]. The goals of the previous studies were thus different from that of CRFref, which aims at identifying highly related references about gene-disease associations, rather than extracting and classifying sentences in the references. On the other hand, to identify references about specific entities (chemicals, genes, or diseases), previous systems often preferred those abstracts in which the entities appeared frequently in the abstracts [33, 34], appeared at certain positions (e.g., the titles, the first sentences, and the last sentences of the abstracts [33, 34]), or co-occurred in a sentence [18, 34]. CRFref aims at identifying those references that expert curators select to develop the summaries for specific gene-disease associations. Such references are highly related to the gene-disease pairs, rather than simply about the gene-disease pairs. Therefore, CRFref considers the degrees of conclusiveness, richness, and focus of each reference, while the previous systems did not simultaneously consider the three degrees. We will show that CRFref performs significantly better than a position- and frequency-based technique in identifying highly related references for over one thousand gene-disease pairs.

## Methods

The research method of the paper consists of two phases: the development phase and the evaluation phase in which CRFref is developed and evaluated respectively.

### Development of CRFref

The input of CRFref includes a reference *r*, a gene-disease pair < *g*, *d*>, and the thesauri of gene names and disease names (with their synonyms). The output of CRFref is the score of *r* with respect to < *g*, *d*>. CRFref is developed to work on the title and the abstract of *r* (rather than the main text of *r*), as titles and abstracts of references are publicly available and convey the main idea of the references.

The thesauri of genes names and disease names are required, as the estimation of the degrees of conclusiveness, richness, and focus of *r* require the recognition of *g* (and its aliases), *d* (and its aliases), and those genes and diseases that are neither *g* nor *d* (as noted in Section Problem definition and main contribution). To setup the set of gene names, we employ the database provided by HGNC (HUGO Gene Nomenclature Committee), which has comprehensively assigned symbols (and names) to human genes [35]. All gene symbols and names assigned by HGNC are downloaded from the website of HGNC^*i*^. To setup the set of disease names, we employ 2013 MeSH. As we are concerned with genetic disorders, we employ all MeSH terms under the MeSH tree nodes C04 to C26, but exclude ‘disease’, ‘syndrome’ and all terms under C01 (Bacterial Infections and Mycoses), C02 (Virus Diseases), and C03 (Parasitic Diseases) as they are not related to genetic disorders^*j*^.

We develop a set of numerical factors to estimate the degrees of conclusiveness, richness, and focus. The resulting design of the factors is presented in Section Development of CRFref. To integrate the factors, we employ RankingSVM [20], which is one of the best techniques routinely used to integrate multiple factors with SVM (Support Vector Machine) to achieve better ranking (e.g., [23, 25]). We employ SVM^*rank*^ to implement RankingSVM^*k*^.

### Evaluation of CRFref

Experiments are designed to empirically evaluate the contributions of CRFref. We conduct comprehensive evaluation on a broad coverage of diseases and genes. Table 1 summarizes main settings of the experiments, which are described in the following subsections.

**Table 1:** **Experimental setup for evaluating CRFref**

Item	Setting
Experimental Data	(1) Gene-disease pairs and candidate references:
	(A) *Genes, diseases, and their aliases*: All diseases (and their aliases) listed in GHR as well as their associated genes (and their aliases) are downloaded.
	(B) *Candidate references*: Abstracts of references for each of the gene-disease pairs are collected by querying PubMed.
	(C) *Target gene-disease pairs*: Target gene-disease pairs are obtained from GHR.
	(2) Target references of each target gene-disease pair:
	For each target gene-disease pair, all candidate references that GHR curators employed to develop a summary for the pair are the target references for the pair.
Baselines	(1) Vector Space Model (VSM) and term weighting techniques: Two techniques: *Lucene* and *BM25*;
	(2) Proximity-based techniques: Two techniques *PRE* and *PLM* that enhance *BM25* with the proximity of the disease and the gene in the references;
	(3) Position-and-frequency-based technique: A technique *PosFreq* that ranks candidate references by considering the positions and the frequencies of the disease and the gene in the references;
	(4) Integrative techniques: Several rankers developed by combining the above techniques with SVM^rank^.
Evaluation criterion	(1) *Mean Average Precision* (MAP);
	(2) *Average Precision at top-X* (average P@X), with X set to 1, 2, and 3.

#### The data

A ranking technique can be used to support the curation and analysis of *new* gene-disease associations reported in literature if it can rank high those references that expert curators have employed to edit the information about specific gene-disease associations. Therefore, in the experiment we evaluate how CRFref (and several baseline techniques) can rank high those references that curators of GHR have employed to develop the summaries for gene-disease associations. We thus consider all diseases (and their aliases) listed in GHR as well as their associated genes (and their aliases)^*l*^. For each gene-disease pair < *g*, *d* > noted by GHR, candidate references are collected by querying PubMed with the filter of ‘Medical Genetics’^*m*^. The form of the query sent to PubMed is as follows:

Medical Genetics [Filter] AND (d and its aliases in disjunctive form) AND (g and its aliases in disjunctive form) AND (“0001”[Date - Publication] : “δ”[Date - Publication])

In the query form, “0001” and “δ” are used to set the range of the years of publication of the references (i.e., from year 0001 to year δ). We set δ to the year of publication of the most-recent reference that GHR curators employed to develop a summary for < *g*, *d*>. By the query we can focus on the references that have been evaluated by GHR curators, although there are gene-disease pairs (in GHR) for which references selected by GHR curators cannot be retrieved by the query^*n*^. We thus obtain 850,605 references pertaining to topics in medical genetics for 1,240 gene-disease pairs (for 664 diseases and 1,006 genes)^*o*^. For each gene-disease pair, we focus on the references whose abstracts mention both the gene and the disease^*p*^, because such references are likely to be about the associations between the gene and the disease. We thus totally have 101,899 such references, which serve as the candidate references in the experiment.

**Figure 1:**
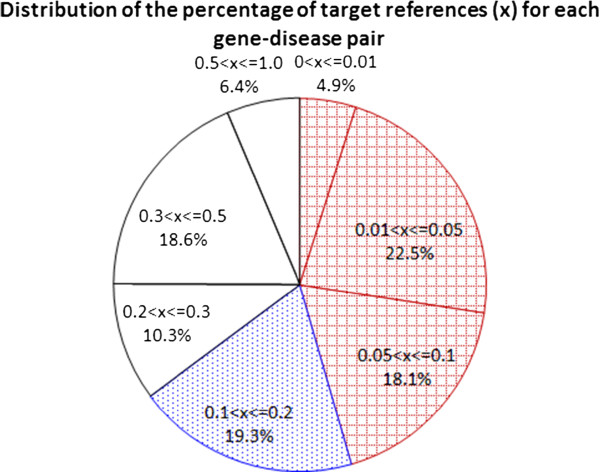
**Distribution of the percentage of target references for each gene-disease pair: The percentage distributes diversely, indicating that the rankers have diverse degrees of difficulty in ranking the target references high for the gene-disease pairs.**

For each gene-disease pair, GHR curators may select some references that are about the gene or the disease but not both. Therefore, not all references selected by GHR for the gene-disease pair can be retrieved as candidate references, which need to mention both the gene and the disease. Therefore, for each target gene-disease pair, all candidate references that GHR curators employed to develop a summary for the pair are the target references for the pair^*q*^. We thus obtain 4,349 target references. For each gene-disease pair that has target references, we analyze the percentage of the target references in the list of candidate references. Figure 1 shows that the percentage distributes diversely, which indicates that the rankers have diverse degrees of difficulty in ranking the target references high for the gene-disease pairs. All terms in the references are normalized by replacing all non-alpha-numeric characters with a space character and replacing upper-case characters with lower-case characters except for those terms that consist of upper-case characters.

Since CRFref and the baseline techniques require training data to build their rankers, we evenly split the 1,240 target gene-disease pairs into four parts. A 4-fold cross validation is then conducted. The data is evenly split into four parts. In each experiment fold, a part of the data is used for testing while the other three parts are used for training. The cross-validation process is then repeated four times, with each of the four parts used exactly once as the testing data. We then report the average performance of CRFref and each baseline, and conduct two significance tests (the paired *t*-test and the Wilcoxon signed-rank test) to verify whether the performance differences between CRFref and the baselines are statistically significant. A performance difference is statistically significant only if both tests show that they are statistically significant.

#### Baseline techniques for performance comparison

To evaluate CRFref with respect to typical ranking techniques, we implement four types of rankers as the baselines: (1) rankers based on Vector Space Model (VSM) and term weighting techniques, (2) proximity-based rankers, (3) position-and-frequency-based rankers, and (4) integrative rankers that integrate multiple rankers. All the rankers receive a candidate reference *r* and a query *q*, which is a gene-disease pair < *g*, *d* > with aliases of *g* treated as *g* and aliases of *d* treated as *d*. The rankers return a score for *r* so that all the candidate references are sorted based on their scores.

For the first type of baselines, we test two popular rankers: Lucene and BM25. Lucene was often employed in previous biomedical text retrieval and mining studies (e.g., [33]). It estimates similarities between a query and a document based on VSM, which is a popular technique to measure query-document similarity. We employ Apache Lucene 4.4 in the experiment^*r*^. On the other hand, BM25 [36] was a popular baseline in many previous studies as well [19]. It ranks a reference *r* higher if *r* shares more highly-weighted terms with the query *q*, with a special consideration on the mean length of the references. More specifically, the BM25 score of *r* with respect to *q* is measured by Equation 1.
1

In Equation 1, N is the total number of training references, *n* is the number of training references containing *t* (either *g* or *d* in the given gene-disease pair), *k*_1_ = 2, *b* = 0.75, *|r*| is the number of words in *r* (length of *r*), *avgrl* is the average number of words in training references (average length of training references), and TF is the term frequency. Similar ideas of Lucene and BM25 have been employed by many search engines as well.

For the second type of baselines, we implement two techniques that enhance BM25 by the proximity between genes and diseases in the references. The two techniques are PRE (**P**roximity-based **R**anker **E**nhancer [25]) and PLM (**P**roximity **L**anguage **M**odel, [27]). They were shown to be two of the best proximity-based techniques to enhance text rankers [25, 27]. They are applied to BM25 by adjusting the TF of the gene and the disease (recall that BM25 has a TF factor, ref. Equation 1). Given a reference *r* and a query *q*, both PRE and PLM assess the term frequency (TF) of *t* (either *g* or *d* in the query) in *r*, and expect that BM25 may be improved if it employs the adjusted TF. Both PRE and PLM assign a larger increment to TF of *t* if other terms in *q* appear at a nearby area of *t* in *r*. The incremented TF helps BM25 to assign a larger score to *r*. For more detailed definitions of PRE and PLM, the reader is referred to [25] and [27], respectively.

For the third type of baselines, we implement a ranker PosFreq based on the idea of eGRAB (Extractor of Gene-Relevant ABstracts), which retrieved those abstracts in which the gene appeared at the titles, the first sentences, and the last sentences of the abstracts, as well as those abstracts in which the gene appeared at least three times [33]. Given a gene-disease pair < *g*, *d* > and a reference *r*, PosFreq employs eight factors: (1) whether *g* appears in the title of *r*, (2) whether *d* appears in the title of *r*, (3) whether *g* appears at the first sentence of the abstract of *r*, (4) whether *d* appears at the last sentence of the abstract of *r*, (5) whether *g* appears at the last sentence of the abstract of *r*, (6) whether *d* appears at the first sentence of the abstract of *r*, (7) whether *g* appears at least three times in the abstract of *r*, and (8) whether *d* appears at least three times in the abstract of *r*. To integrate the eight factors, we employ RankingSVM, which is one of the best techniques to integrate multiple factors (as noted in Section Development of CRFref). PosFreq aims at representing those techniques that retrieves references based on both the positions and the frequencies of biomedical entities in the references (as noted in Section Text ranking technology).

For the fourth type of baselines, we create several integrative rankers by combining the above baselines with RankingSVM. By comparing the performance of CRFref and the integrative rankers, we can evaluate CRFref more comprehensively.

#### Evaluation criteria

We employ *mean average precision* (MAP) and *average precision at top-x* (average P@X) as two criteria to evaluate the performance of each ranker. Both criteria were commonly employed to evaluate text rankers. They aim at measuring how highly related references are ranked higher for the curators to read. A ranker that can achieve better MAP and average P@X is thus expected to be more capable of providing more references that deserve consideration by the curators, even though the curators have not selected the references. In practice, such rankers can be a good tool for the curator to build the database of gene-disease associations. More specifically, MAP is defined in Equation 2.
23

In Equation 2, |*Q*| is the number of gene-disease pairs, and hence MAP is the average of the average precision (AP) values for all gene-disease pairs. The AP value for the *i*^th^ gene-disease pair is defined in Equation 3, where *k*_*i*_ is the number of target references for the *i*^th^ gene-disease pair, and *Doc*_*i*_(*j*) is the number of references whose ranks are higher than or equal to that of the *j*^th^ target reference for the *i*^th^ gene-disease pair. If a ranker can rank higher the target references (i.e., those that have been employed by GHR curators to develop a summary for the gene-disease pair), the AP value for the pair will be larger.

On the other hand, average P@X measures how each ranker ranked the target references at the top-X positions. It is defined in Equation 4.
45

In Equation 4, |*Q*| is the number of gene-disease pairs, and hence average P@X is the average of the P@X values for all gene-disease pairs. The P@X value for the *i*^th^ gene-disease pair is defined in Equation 5, which measures the precision rate when top-X references are seen by the reader. By setting X to a small value, P@X can be used to measure whether a ranker can rank target references very high so that expert curators can focus on reading only a small number of recommended references. We thus report average P@X, with X set to 1, 2, and 3.

## Result

### Development of CRFref

Table 2 defines the thirteen factors that CRFref employs to measure the degrees of *conclusiveness* (ref. the 1^st^ to the 7^th^ factors), *richness* (ref. the 8^th^ to the 9^th^ factors), and *focus* (ref. the 10^th^ to the 13^th^ factors) of a reference *r* with respect to a gene-disease pair < *g*, *d*>. With the aliases of genes and diseases noted in GHR, aliases of *g* are treated as *g*, and aliases of *d* are treated as *d*. The thirteen factors are integrated by RankingSVM (as noted in Section Development of CRFref). In the 4-fold cross validation experiments (ref. Section The data), training data is used to train RankingSVM, which is then tested with the test data.

**Table 2:** **Given a reference** ***r*** **and a gene-disease pair <** ***g*** **,** ***d*** **>, CRFref estimates and integrates three measures: degrees of** ***conclusiveness*** **,** ***richness*** **, and** ***focus*** **of** ***r*** **with respect to <** ***g*** **,** ***d*** **>**

Factors	Definition	Type
(1) Length(*r*)		*Degree of conclusiveness*
(2) GeneTF(*g*,*r*)		
(3) DiseaseTF(*d*,*r*)		
(4) Gene@Title(*g*,*r*)		
(5) Disease@Title(*d*,*r*)		
(6) Gene@Ending(*g*,*r*)		
(7) Disease@Ending(*d*,*r*)		
(8) NotGeneNum(*g*,*r*)		*Degree of richness*
(9) NotDiseaseNum(*d*,*r*)		
(10) NotGene@Title(*g*,*r*)		*Degree of focus*
(11) NotDisease@Title(*d*,*r*)		
(12) NotGene@Ending(*g*,*r*)		
(13) NotDisease@Ending(*d*,*r*)		

^[*a*]^ *AvgLen* is the average length of references.

^[*b*]^ *TF*(*x*,*r*): Term frequency of *x* in *r.*

^[*c*]^ *LastPos*(*x*,*r*): The last position of *x* in *r.*

^[*d*]^ *G*: Set of gene names in HUGO Gene Nomenclature Committee (HGNC).

^[*e*]^ *D*: Set of terms in MeSH class of C04 to C26, with ‘disease’ and ‘syndrome’ removed.

The 1^st^ to the 3^rd^ factors are commonly employed by text rankers. The 1^st^ factor (Length) is the normalized length of *r* (i.e., normalized number of words in *r*). The normalization is conducted by employing the average length (*AvgLen*) of the references in the training data so that Length(*r*) is set to 1.0 if length of *r* is greater than *AvgLen*. The factor is related to the “density” of *g* and *d* in *r* (a smaller Length(*r*) tends to increase the density), and hence is related to the degree of conclusiveness of *r* with respect to < *g*, *d*>. The 2^nd^ factor (GeneTF) and the 3^rd^ factor (DiseaseTF) count the term frequencies of *g* and *d* in *r*, respectively. They are related to the degree of conclusiveness as well, since they indicate how *r* repeatedly mentions *g* and *d*. GeneTF and DiseaseTF are normalized, and hence they are in the range of [0, 1].

The 4^th^ to the 7^th^ factors are more related to the degree of *conclusiveness*. The 4^th^ factor (Gene@Title) and the 5^th^ factor (Disease@Title) are concerned with whether *g* and *d* appear in the title of *r* respectively. If *g* and *d* appear in the title of *r*, we expect that *r* provides conclusive results about the association between *g* and *d*. Another “concluding” part of *r* that might provide conclusive results should be the ending part of *r*, as authors of a reference tend to provide their research conclusions at the end of the reference. Therefore, the 6^th^ factor (Gene@Ending) and the 7^th^ factor (Disease@Ending) transform the last position of *g* and *d* in *r* into a score respectively. The score is normalized by the length of *r*. It will be quite small if the last position is far from the end of *r*.

The 8^th^ factor (NotGeneNum) and the 9^th^ factor (NotDiseaseNum) aim at measuring the degree of *richness* of *r* with respect to < *g*, *d*>. NotGeneNum is concerned with the number of distinct genes that are not *g* but appear in *r*. Similarly, NotDiseaseNum is concerned with the number of distinct diseases that are not *d* but appear in *r*. Therefore the two factors consider whether *r* mentions several genes and diseases that are *neither g nor d* but are related to *g* and *d*. In that case, *r* might provide rich information about < *g*, *d*>, and hence its degree of richness for < *g*, *d* > may be larger. As noted in Section Development of CRFref, for the thesaurus of gene names (i.e., the set *G* in Table 2) we employ the database provided by HGNC, and for the thesaurus of disease names (i.e., the set *D* in Table 2) we employ 2013 MeSH.

The 10^th^ to the 13^th^ factors aim at measuring the degree of *focus* of *r* with respect to < *g*, *d*>. They are actually the counterparts of the 4^th^ to the 7^th^ factors respectively − they are concerned with how *r* provides *conclusive* results about those genes and diseases that are *neither g nor d*. Therefore, the four factors are concerned with whether such genes and diseases appear in the title (ref. the 10^th^ factor NotGene@Title and the 11^th^ factor NotDisease@Title) and the ending part (ref. the 12^th^ factor NotGene@Ending and the 13^th^ factor NotDisease@Ending) of *r*. We expect that if *r* provides conclusive results about such genes and diseases, *r* might *not* focus on the association between *g* and *d*.

### Evaluation of CRFref

#### CRFref and each individual baseline

**Figure 2:**
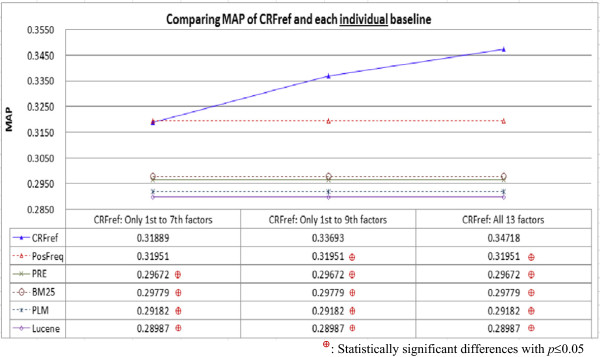
**Comparing MAP of CRFref and each individual baseline: When CRFref only considered the degree of conclusiveness, it had been able to perform significantly better than all baselines except for PosFreq, which had quite similar performance; When CRFref considered conclusiveness, richness, and focus, it performed significantly better than all the baselines.**

Figure 2 compares MAP of CRFref and individual baselines, which include the first three types of rankers noted in Section Baseline techniques for performance comparison (i.e., PosFeq, PRE, BM25, PLM, and Lucene). To verify whether the performance differences were statistically significant, we conducted two significance tests (the Wilcoxon signed-rank test and the *t*-test) on the AP values for the 1,240 gene-disease pairs. A performance difference was statistically significant only if both tests showed that they were statistically significant. The results showed that when CRFref only considered the degree of conclusiveness (i.e., employing only the 1^st^ to the 7^th^ factors defined in Table 2), it was able to perform significantly better than all baselines except for PosFreq. The performance difference between PosFreq and the “conclusiveness-only” version of CRFref was not statistically significant. This result confirms that the degree of conclusiveness is a key concern for expert curators to select biomedical references to develop summaries for gene-disease associations. Term proximity (mainly considered by PRE and PLM) and term weights (mainly considered by BM25 and Lucene) are *not* the key factors in identifying highly related references about the gene-disease associations.

**Figure 3:**
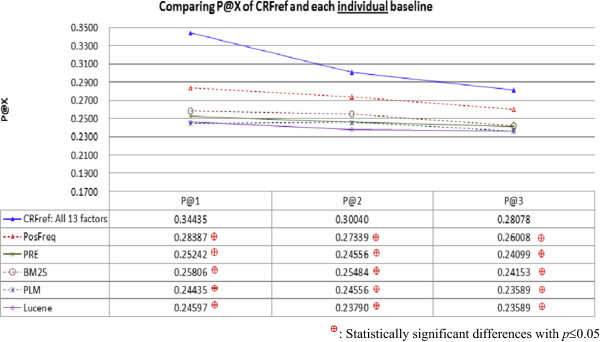
**Comparing average P@X of CRFref and each individual baseline: CRFref performed significantly better than all the baselines, indicating that CRFref ranked highly related references very high so that expert curators can focus on reading only a small number of recommended references.**

Moreover, when CRFref considered both conclusiveness and richness (i.e., using the 1^st^ to the 9^th^ factors defined in Table 2), it performed significantly better than all the baselines, including PosFreq. This result confirms the contribution of the degree of richness. When CRFref considered conclusiveness, richness, and focus (i.e., using all the 13 factors in Table 2), it performed even better. This result confirms the contribution of the degree of focus, which helps to rank lower those references that provide *conclusive* information about many genes and diseases that are *neither* the target gene *nor* the target disease. The degree of focus is actually an extension of the degree of conclusiveness, and with the extension CRFref performs even better.Figure 3 compares average P@X of CRFref and the individual baselines. Similarly, to verify whether the performance differences were statistically significant, two significance tests were conducted on the P@X values for the 1,240 gene-disease pairs. The results showed that CRFref performed significantly better than all the baselines, indicating that CRFref ranked highly related references very high (top-3) so that expert curators can focus on reading only a small number of recommended references. With the better results in both MAP and average P@X, CRFref is expected to be more capable of providing more references that may deserve consideration by the curators, even though the curators have not selected the references (this may be a reason why P@X for each gene-disease pair is not necessarily high in the experiment).

We also analyzed the percentage of gene-disease pairs for which P@X > 0. A higher percentage indicates that the ranker can help expert curators to read a small number of recommended references for more gene-disease pairs. Table 3 shows the result. CRFref ranked very high (top-3) the target references for over 53% of the gene-disease pairs, while all the baselines did not achieve the high percentage. The contribution is of practical significance to curators, because there are often a large number of gene-disease pairs, and each pair often has a large number of candidate references to be checked by the curators.

**Table 3:** **Percentage of gene-disease pairs for which P@X > 0: When compared with the individual baselines, CRFref ranked highly related references at top-3 for a higher percentage of gene-disease pairs**

Ranker	% of pairs for which P@1 > 0	% of pairs for which P@2 > 0	% of pairs for which P@3 > 0
CRFref	34.44%	46.29%	53.55%
PosFreq	28.39%	40.81%	47.90%
PRE	25.24%	36.13%	44.44%
BM25	25.81%	37.50%	44.76%
PLM	25.56%	37.02%	44.60%
Lucene	24.60%	35.40%	43.79%

#### CRFref and the integrative baselines

We further investigated whether CRFref performed better than the integrative baselines (i.e., the fourth type of baselines noted in Section Baseline techniques for performance comparison), which were implemented by integrating the above individual rankers with RankingSVM. Since PosFreq was the best baseline, we integrated it with the other four baselines PRE, BM25, PLM, and Lucene, and the resulting integrative rankers were named PRE + PosFreq, BM25 + PosFreq, PLM + PosFreq, and Lucene + PosFreq, respectively.

**Figure 4:**
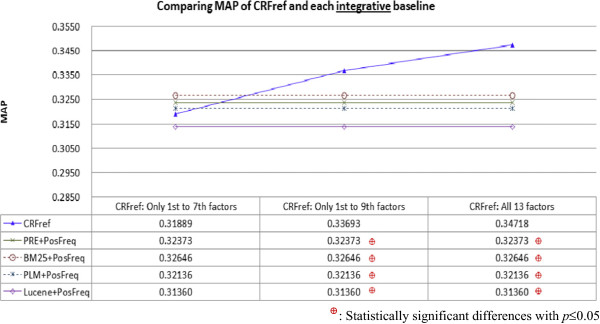
**Comparing MAP of CRFref and each integrative baseline: Although each baseline was improved by integrating it with PosFreq, CRFref performed significantly better than all the integrative baselines when it considered conclusiveness, richness, and focus.**

Figure 4 compares MAP of CRFref and the integrative rankers. The result indicated that the integration was often helpful for the individual baselines. Most integrative rankers (i.e., PRE + PosFreq, BM25 + PosFreq, and PLM + PosFreq) performed better than PosFreq (ref. performance of PosFreq shown in Figure 2 and performance of the integrative rankers shown in Figure 4), indicating that PosFreq benefited from the integration as well. Although some of the integrative rankers performed better than “conclusiveness-only” version of CRFref, all the performance differences were *not* statistically significant. When CRFref considered conclusiveness, richness, and focus, it performed significantly better than all the integrative rankers. This result reconfirms the contribution of CRFref.We also implemented a ranker by integrating all the individual rankers (PRE, BM25, Lucene, and PosFreq) except for PLM (since both PLM and PRE contributed term proximity information, and the results shown in Figures 2 and 3 indicated that PRE performed better than PLM). MAP of the ranker was 0.31671, which was lower than the MAP of most integrative rankers. Therefore, integration of many baselines does not necessarily produce better performance.

**Figure 5:**
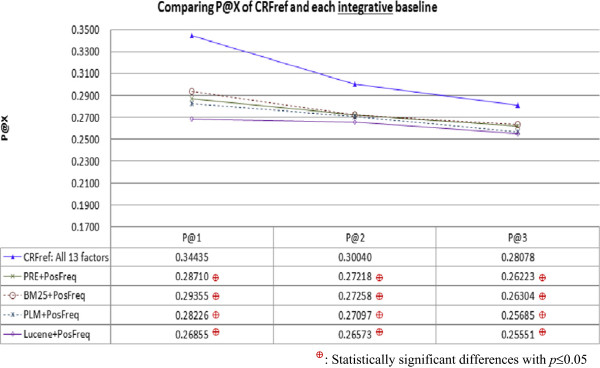
**Comparing average P@X of CRFref and each integrative baseline: Although each baseline was improved by integrating it with PosFreq, CRFref performed significantly better than all the integrative baselines.**

Figure 5 compares average P@X of CRFref and the integrative rankers. Again, CRFref performed significantly better than all the rankers, even though they were improved by integration with PosFreq. Table 4 shows the percentage of gene-disease pairs for which P@X > 0. Again, CRFref achieved a higher percentage than all the integrative baselines. With the support provided by CRFref, expert curators can check a small number of recommended references for more gene-disease pairs. This results reconfirm the contribution of CRFref.

**Table 4:** **Percentage of gene-disease pairs for which P@X > 0: When compared with the integrative baselines, CRFref ranked highly related references at top-3 for a higher percentage of gene-disease pairs**

Ranker	% of pairs for which P@1 > 0	% of pairs for which P@2 > 0	% of pairs for which P@3 > 0
CRFref	34.44%	46.29%	53.55%
PRE + PosFreq	28.71%	41.85%	50.24%
BM25 + PosFreq	29.35%	41.77%	50.32%
PLM + PosFreq	28.23%	41.94%	48.47%
Lucene + PosFreq	26.85%	40.32%	47.98%

#### Integration of CRFref and the baselines

We are also interested in the possible contribution of integrating CRFref and each of the individual baselines and the integrative baselines. The integration was achieved by RankingSVM as well. Table 5 compares MAP values of CRFref and those baselines that were integrated with CRFref. By the integration with CRFref, all individual baselines and integrative baselines were improved. However, all the integrated versions did not perform significantly better than CRFref, and CRFref even performed better than some of them. For example, although CRFref + Lucene performed much better than Lucene, it performed significantly worse than CRFref. This result indicates that CRFref has been a good ranker that can be used independently. Other kinds of factors concerning term frequencies, term positions, term proximity, and term weights are not necessarily helpful in improving CRFref.

**Table 5:** **Comparing MAP of CRFref and the rankers constructed by integrating the baselines and CRFref: All baselines were improved by integrating them with CRFref, however all the integrated versions did not perform significantly better than CRFref**

Type	Ranker	MAP
CRFref	CRFref	0.34718
CRFref & individual baselines	CRFref + PosFreq	0.35046
	CRFref + PRE	0.34447
	CRFref + BM25	0.34855
	CRFref + PLM	0.34630
	CRFref + Lucene	0.34138^s^
CRFref & integrative baselines	CRFref + PRE + PosFreq	0.35010
	CRFref + BM25 + PosFreq	0.35231
	CRFref + PLM + PosFreq	0.34974
	CRFref + Lucene + PosFreq	0.34639

^s^: Statistically significant differences with *p* ≤ 0.05

Table 6 compares average P@X of CRFref and the integrated versions. All baselines were improved by integrating them with CRFref. When compared with CRFref, some of the integrated versions achieved better performance in P@3 but not P@1.

**Table 6:** **Comparing average P@X of CRFref and the rankers constructed by integrating the baselines with CRFref: All baselines were improved by integrating them with CRFref, however when compared with CRFref, some of the integrated versions achieved better performance in P@3 but not P@1**

Type	Ranker	P@1	P@2	P@3
CRFref	CRFref	0.34435	0.30040	0.28078
CRFref & individual baselines	CRFref + PosFreq	0.33065	0.30202	0.28965
	CRFref + PRE	0.32581^s^	0.30161	0.28777
	CRFref + BM25	0.33548	0.30323	0.28938^s^
	CRFref + PLM	0.33548	0.30242	0.28589
	CRFref + Lucene	0.33306	0.29516	0.28132
CRFref & integrative baselines	CRFref + PRE + PosFreq	0.32258^s^	0.30685	0.29073^s^
	CRFref + BM25 + PosFreq	0.33629	0.30887	0.29019
	CRFref + PLM + PosFreq	0.32500^s^	0.30524	0.29341^s^
	CRFref + Lucene + PosFreq	0.33468	0.30161	0.28159

^s^: Statistically significant differences with *p* ≤ 0.05

#### A case study

After showing that CRFref performed better than all the baselines, we conducted a case study to further illustrate the contribution of CRFref. The case study was about the disease *sickle cell disease*. GHR experts noted that *sickle cell disease* was associated with the gene *beta-globin*, which was associated with three disorders, including *sickle cell disease*, *beta-thalassemia*, and *beta-globin type methemoglobinemia*. The GHR experts selected 8 references to develop the summary for *sickle cell disease*^*t*^. The references were about the overview, the risk factor, the outcome, and the therapy of *sickle cell disease*. Among the 8 references, only one citation (PubMed ID: 10791557) was about *sickle cell disease* and *beta-globin*, and hence was the target reference selected to develop the summary for the association between *sickle cell disease* and *beta-globin*. We were thus concerned with the cost of identifying the target reference using PubMed, the best baselines, and CRFref. The cost was an indicator to measure the contribution of CRFref to the curation of the association between *sickle cell disease* and *beta-globin*.

The query sent to PubMed was in the form noted in Section The data, with the aliases of *sickle cell disease* and *beta-globin* being added to the query^*u*^. PubMed returned 330 references. It employed the publication date of each reference as a factor to rank the 330 references^*v*^. Table 7 shows how the target reference and a non-target reference (PubMed ID: 12972407) were ranked by PubMed, CRFref, and the best baselines (BM25 + PosFreq and PRE + PosFreq). We considered the non-target reference because the two best baselines ranked it at the top-1 position. PubMed and the baselines preferred the non-target reference to the target one. It ranked the target reference at the 34^th^ position, indicating that for a curator that employed PubMed to identify references to curate the association between *sickle cell disease* and *beta-globin*, 33 references needed to be read and checked, which would be a heavy burden for the curator. CRFref ranked the target reference at top-3, which should be helpful for the curator.

**Table 7:** **Two references ranked by PubMed, CRFref, and the best baselines (BM25 + PosFreq and PRE + PosFreq): CRFref ranked the target reference at top-3, while PubMed and the baselines preferred the non-target reference**

Reference	Position			
	PubMed	BM25 + PosFreq	PRE + PosFreq	CRFref
Target reference (PubMed ID: 10791557)	34^th^	5^th^	6^th^	3^rd^
Non-target reference (PubMed ID: 12972407)	13^th^	1^st^	1^st^	6^th^

PubMed ranked the target reference at the 34^th^ position, indicating that for an expert that employed PubMed to identify references to curate the gene-disease association, 33 references needed to be read and checked, which was a burden for the expert.

We further analyze the target reference and the non-target reference. The target reference (excerpted) is as follows:

*PubMed ID*: 10791557 (by Ashley-Koch A, Yang Q, Olney RS, published in *Am J Epidemiol*.)

*Title*: *Sickle hemoglobin (HbS) allele and sickle cell disease: a HuGE review.*

Abstract: *Sickle cell disease* is caused by a variant of the *beta-globin* gene called sickle hemoglobin (Hb S). … either two copies of Hb S or one copy of Hb S plus another *beta-globin* variant (such as Hb C) are required for disease expression. … individuals with *sickle cell disease* exhibit significant morbidity and mortality. Symptoms include chronic anemia, acute chest syndrome, stroke, … Disease expression is variable and is modified by several factors, the most influential being genotype. Other factors include *beta-globin* cluster haplotypes, alpha-globin gene number, and fetal hemoglobin expression. … newborn screening, better medical care, parent education, and penicillin prophylaxis have successfully reduced morbidity and mortality due to Hb S.

The target reference focuses on the disease (*sickle cell disease*) and its association with the gene (*beta-globin*), although the reference does not mention the gene and the disease many times. CRFref successfully ranked the reference at top-3, based on two reasons: (1) the gene and the disease appear at the concluding parts of the reference (i.e., the title and the ending part), and (2) the reference tends not to mention other genes and diseases at the concluding parts. On the other hand, the non-target reference (excerpted) is as follows:

*PubMed ID*: 12972407 (by Pritchard KA Jr, Ou J, Ou Z, Shi Y, Franciosi JP, Signorino P, Kaul S, Ackland-Berglund C, Witte K, Holzhauer S, Mohandas N, Guice KS, Oldham KT, Hillery CA., published in *Am J Physiol Lung Cell Mol Physiol*.)

*Title*: *Hypoxia-induced acute lung injury in murine models of sickle cell disease.*

*Abstract*: Vaso-occlusive events are the major source of morbidity and mortality in *sickle cell disease* (*SCD*); however, the pathogenic mechanisms driving these events remain unclear. Using hypoxia to induce pulmonary injury, we investigated mechanisms by which sickle hemoglobin increases susceptibility to lung injury in a murine model of *SCD*, where mice either exclusively express the human alpha/sickle *beta-globin* (halphabetaS) transgene (*SCD* mice) or are heterozygous for the normal murine *beta-globin* gene and express the halphabetaS transgene (mbeta+/-, halphabetaS+/-; heterozygote *SCD* mice). … Hypoxia further decreased association of HSP90 with eNOS in lungs of *SCD* and heterozygote *SCD* mice, but not in the control lungs. … These data support the hypotheses that hypoxia increases XO release from ischemic tissues and that the local increase in XO-induced oxidative stress can then inhibit HSP90 interactions with eNOS, decreasing *NO generation and predisposing the lung to vaso-occlusion.

The non-target reference does not focus on the association between the disease and the gene. It is about the pathogenic mechanisms that drive vaso-occlusive problems in murine models of sickle cell disease. The reference is preferred by PubMed and the best baselines, simply because of two reasons: (1) the gene and the disease appear many times and are near to each other, and (2) the disease appears at the title. CRFref successfully ranked the non-target reference lower because several genes (e.g., XO and eNOS) other than *beta-globin* appear in a concluding part (i.e. end of the abstract) of the reference, making the reference less focused on the *sickle cell disease* and *beta-globin*.

## Discussion

### Application and suggestion

The results have shown that existing rankers, which rank references by typical factors (i.e., term frequencies, term weights, term proximity, and term positions), perform significantly poorer than CRFref in identifying those references that expert curators employ to develop summaries for specific gene-disease associations. CRFref can thus serve to identify highly related references for the curation of gene-disease associations published in literature. Given a gene-disease pair, the curators can retrieve candidate references by whatever ways (e.g., expanding the queries by related terms) and constraints (e.g., limiting the publication time of the references). CRFref can then be used to prioritize the candidate references to support more timely and complete curation. CRFref can also serve as a front-end processor for many text mining systems so that the systems can focus on highly related references, and hence promotion of the quality of the mining results may be expected.

Another interesting application of CRFref is the identification of the highly related references about the interactions and associations between different biomedical entities, such as proteins, genes, chemicals, drugs, and diseases. We expect CRFref may perform well in the application since the main idea of CRFref is not limited to the associations between genes and diseases. As biomedical researchers are often concerned with the interactions and associations between different entities, CRFref can help the researchers by recommending highly related references to them.

To implement the above applications, we suggest that CRFref should be employed as a component in literature search engines (e.g., PubMed). This is because the measurement of conclusiveness, richness, and focus is helpful, but it cannot be efficiently done to support online reference retrieval if the references in the database are not pre-processed. The preprocessing should include the identification of the locations of the biomedical entities in the references. The preprocessed and cached location information makes the search engines able to efficiently measure conclusiveness, richness, and focus. The ideas of CRFref should thus be incorporated into the archival and ranking functions of the existing search engines so that highly related references can be ranked higher for the users online.

### Limitation and future research

The data collection strategy has limitations. The candidate references for a gene-disease pair < *g*, *d* > are those references that mention both *g* and *d* (and their aliases). This way of data collection is reasonable, as we aim at identifying highly-related references for < *g*, *d*>. However, not all references retrieved by PubMed can be candidate references (850,605 were retrieved by PubMed but among which only 101,899 were candidate references). This was because PubMed expanded and relaxed the query to retrieve more references (e.g., expanding the query by related terms, and relaxing the query by breaking a phrase into individual terms)^*s*^. We expect that the references retrieved by a relaxed query should tend to be *not* highly related to < *g*, *d*>. However, the effect of such references is not explored in the paper.

Another limitation of our data collection strategy is that for some gene-disease pairs in GHR, the references selected by GHR experts cannot be retrieved by the query noted in Section The data (there are 480 such pairs, among the 1,720 pairs in GHR). Such pairs are excluded and hence they are not tested by the ranking systems in the experiment, although all the ranking systems have worked on the same set of gene-disease pairs (i.e., 1,240 pairs). To retrieve more references, the query may be relaxed by removing some constraint (e.g., removing the “medical genetics” filter) from the query or incorporating more aliases of the gene and the disease in the query.

CRFref has technical limitations as well, which deserve tackling in future research. Given a gene-disease pair < *g*, *d*>, the degree of focus of a reference is based on how the reference provides conclusive information about the genes and the diseases that are neither *g* nor *d*. To identify such genes and diseases, CRFref employs thesauri of gene names and disease names. This way of identification cannot properly deal with the common cases where *d* might have several related but different diseases or syndromes. For example, consider the target reference discussed in the above case study. This reference focuses on the gene-disease pair < *beta-globin*, *sickle cell disease* > but it also mentions several “symptoms” of the target disease ‘*sickle cell disease*’, such as ‘*chronic anemia*’, ‘*acute chest syndrome*’, and ‘*stroke*’, which were identified as diseases by CRFref as well. CRFref thus thinks the reference is less focused on the target disease. Similar cases occur when several genes that are related to the target gene happen to appear at the concluding parts (i.e., title and ending part) of the reference. Therefore, more intelligent identification of the related genes and diseases can further refine the measurement of the degree of focus. The related genes and diseases may be recognized and excluded by analyzing the semantics of the sentences (e.g., by parsing) and/or checking the relationships noted in a biomedical thesaurus.

Another limitation of CRFref is incurred by the use of term *positions* to measure the degrees of conclusiveness and focus. To measure how a reference *r* provides conclusive information about the target gene-disease pair < *g*, *d*>, the 6^th^ factor (Gene@Ending) and the 7^th^ factor (Disease@Ending) measure how *g* and *d* appear in the ending part of the abstract of *r*. Similarly, to measure how *r* provides focused information about < *g*, *d*>, the 12^th^ factor (NotGene@Ending) and the 13^th^ factor (NotDisease@Ending) measure how genes and disease other than *g* and *d* appear in the ending part of the abstract of *r*. Although the ending part is likely to be about the conclusion of *r*, the precise position of the conclusion is hard to identify if the abstract is not structured with explicit labels for the conclusion. Therefore, more intelligent identification of the concluding paragraph of a reference is an interesting research problem. Previous techniques that extracted and classified passages (e.g., [37]) may be applicable to the problem. Integration of CRFref and the previous techniques should be helpful in improving CRFref.

## Conclusion

Curation of gene-disease associations published in literature is essential for biomedical research. However, timely and complete curation is both costly and challenging, since the curation needs to be based on careful and frequent survey of the references that are highly related to specific gene-disease associations. Retrieval of the references is thus essential for the curation.

We develop a technique CRFref that, given a gene-disease pair < *g*, *d*>, ranks biomedical references based on how they provide *conclusive*, *rich*, and *focused* results on the associations between *g* and *d*. When identifying the references that expert curators believed to be highly related to specific gene-disease pairs, CRFref performs significantly better than typical types of rankers, which often ranked references based on the weights, proximity, frequencies, and positions of terms. CRFref can thus be incorporated into search engines to prioritize the highly related references to support more timely and complete curation of the gene-disease associations already published in literature.

## Endnotes

^a^Available at http://www.ncbi.nlm.nih.gov/pubmed/clinical.

^b^Available at http://ghr.nlm.nih.gov/.

^c^Available at http://www.ncbi.nlm.nih.gov/omim.

^d^See http://ghr.nlm.nih.gov/about#leading-edge.

^e^See http://ghr.nlm.nih.gov/ExpertReviewers.

^f^See http://www.omim.org/about.

^g^See http://ghr.nlm.nih.gov/whatsnew/show/12months.

^h^GeneWanderer is available at http://compbio.charite.de/genewanderer/GeneWanderer.

^i^117,934 terms were downloaded in July 2013, through http://www.genenames.org/cgi-bin/hgnc_downloads.

^j^45,383 terms were downloaded from 2013 MeSH, which is available at http://www.nlm.nih.gov/mesh/filelist.html.

^k^SVM^rank^ is available at http://www.cs.cornell.edu/People/tj/svm_light/svm_rank.html.

^l^1,720 gene-disease pairs (for 747 diseases and 1,327 genes) were downloaded in November 2012.

^m^The filter of Medical Genetics aims at retrieving references pertaining to topics in medical genetics (available at http://www.ncbi.nlm.nih.gov/pubmed/clinical).

^n^There are 480 pairs (among the 1,720 pairs in GHR) for which references selected by GHR curators cannot be retrieved.

^o^The medical genetics references were retrieved in 11/21 ~ 11/25, 2012, with 100,000 as the maximum number of references returned by PubMed for each query.

^p^For each gene-disease pair, Lucene (available at http://lucene.apache.org) was employed to remove those references that did not mention both the gene and the disease. The removal is reasonable, as we aim at identifying highly-related references for the gene-disease pair (ref. the discussion in Section Limitation and future research).

^q^Among the 1,240 gene-disease pairs, 109 pairs have no target references. This is because for each of the 109 pairs, the references selected by GHR curators are not listed in the candidate references, which need to mention both the gene and the disease. To reduce the amount of such pairs, the existence of a gene and a disease in a reference should be checked by intelligent named entity recognition (rather than strict term matching by Lucene). In the experiment, all the ranking systems work on the 109 pairs as well, because in practice the ranking systems often need to work on those pairs that are not correlated (i.e., have no target references).

^r^Lucene is available at http://lucene.apache.org, and its technique to estimate query-document similarity is outlined at http://lucene.apache.org/core/4_4_0/core/org/apache/lucene/search/similarities/TFIDFSimilarity.html.

^s^The query expansion and relaxing strategies of PubMed can be found at http://www.ncbi.nlm.nih.gov/books/NBK3827/#pubmedhelp.I1_MeSH_translation_.

^t^The list of references that GHR selected to develop the summary for ‘sickle cell disease’ can be found in http://ghr.nlm.nih.gov/condition/sickle-cell-disease/show/References.

^u^The query sent to PubMed was in the form noted in Section The data, with the publication date being set to from year 0001 to year 2005 (since the most recent reference selected for “sickle cell disease” was published in 2005).

^v^The references were sorted by PubMed relevance sort, which considered the frequency and the position of search terms in each reference (as done by the baselines), as well as the publication date of each reference (ref. http://www.nlm.nih.gov/pubs/techbull/so13/so13_pm_relevance.html).
